# A novel model mouth system for evaluation of *In Vitro* release of nicotine from moist snuff

**DOI:** 10.1186/1752-153X-7-176

**Published:** 2013-11-11

**Authors:** Peng Li, Jie Zhang, Shi-Hao Sun, Jian-Ping Xie, Yong-Li Zong

**Affiliations:** 1 Zhengzhou Tobacco Research Institute of China National Tobacco Corporation, Zhengzhou 450001, China; 2 Beijing Workstation of Technology Centre of Shanghai Tobacco (Group) Corporation, Beijing 101121, China

**Keywords:** Model mouth system, Nicotine, In vitro, Release, Moist snuff, Smokeless tobacco products

## Abstract

**Background:**

Pouch moist snuff, as a form of oral smokeless tobacco products, is becoming increasingly popular in North America, Scandinavia (where it is known as Snus), South Asia and parts of Africa. User usually places a pouch between the upper jaw and cheek to obtain euphoria from tobacco, leading to partial intake of tobacco constituents. To evaluate user exposure to tobacco, an approach with a novel model mouth system was developed and applied to evaluate release of nicotine from the pouch.

**Results:**

A novel model mouth system has been developed to evaluate release behavior of tobacco constituents in pouch moist snuff. The system consists of the release medium reservoir module, the flow speed control module, the temperature control module, nicotine release module, and release solution collection module, and simulates buccal condition in terms of temperature, saliva compositions, and the rate of saliva production, etc. Artificial saliva was used as the release medium to evaluate release of nicotine in pouch moist snuff. The optimized test condition was that the release temperature of 37°C and the flow rate performed at 0.2 mL min-1 in the first 5 min and 0.1 mL min-1 in the next 55 min. The performance of the model mouth system was compared with *in vivo* data of nicotine release in human volunteers. Data from 23 brands of moist snuff indicated that nicotine release rates increased with extraction time and approximately 60-90% of nicotine was released after 30 min of extraction in most of the samples, and the release behavior of nicotine was affected by product weights, nicotine concentration, and product pH, etc.

**Conclusion:**

The model mouth system can be used to evaluate the release behavior of constituents in pouch moist snuff, especially those directly related to human health such as nicotine and tobacco specific nitrosamine (TSNA), etc. This indicated that the system is an alternative tool to evaluate user exposure to tobacco. With further testing and validation, the model mouth system can be applied in risk evaluation of smokeless tobacco products.

## Background

Moist snuff is a sub-category of oral smokeless tobacco products (STPs), which is popular in North America, Scandinavia (where it is known as Snus), South Asia (e.g. Bangladesh, Bhutan and India) and parts of Africa (e.g. Algeria, Sudan and Nigeria) [1,2]. It is mainly available in two forms: a loose form of compacted tobacco and a form with portions of the tobacco sealed in small sachets termed “pouches”. A pouch is usually placed between the upper jaw and gum, resulting in absorption of nicotine. It is typically held in the mouth for approximately 30 minutes before discarded. Recently, the “pouches” form is becoming increasingly popular.

Nicotine, a toxic addictive alkaloid, plays a crucial role in maintaining smokeless tobacco use among users [3-6]. And availability of nicotine from a STPs is influenced by a number of factors, such as the weight of the actual unit dose, the pH level of the product, the particle size of the product, the time kept in the mouth, and the tissue material of the pouch [7,8]. The release of nicotine from STPs is an essential first step in nicotine absorption; therefore, the estimation of the extraction of nicotine by users is of particular importance to product safety evaluation and nicotine control of STPs.

*In vitro* release of nicotine from STPs was studied by a couple of investigators previously. Luque-Pérez et al. [9] reported a method using liquid membranes, in which the samples are directly placed in the membrane unit and nicotine released from the samples passes through the membrane (polypropylene/n-undecane) into an acidic acceptor stream. The stream flows through a spectrophotometric detector, allowing the measurement of nicotine absorbance at 260 nm. In a method reported by Nasr et al. [10], samples were placed in dialysis bags soaked in artificial saliva to determine the release rate of nicotine. However, nicotine extraction in these two methods is very different from the buccal condition. So, the applicability of the approaches was restricted by difficulties in reproducing real-use factors such as usage time, mechanical work on the STPs in the mouth of users.

Model mouths are widely used in the food industry to reproduce and study flavor during eating with an emphasis on reproducing the range of phenomena observed such as chewing and swallowing [11-13]. Chewing does not present in the consumption procedure of moist snuff, however, the release of nicotine and other constituents is likely to involve diffusion from moist snuff into the thin layer of saliva coating the mouth. The extractive components from moist snuff are then transferred across the oral mucosa to be absorbed into the bloodstream [14].

A parallel approach to food industry model mouth systems is the use of dissolution apparatus by the pharmaceutical industry to study the release of drugs from dosage forms in the oral cavity such as sublingual tablets and buccal tablets. The methods used for studying the release of these drugs are mainly based on standard dissolution tests in which the tablets are immersed in large amounts of release medium to determine their dissolution rates [15-17].

Methods used in both food and pharmaceutical industry and current methods of nicotine extraction from STPs do not closely simulate the buccal condition of moist snuff consumption. It is therefore desirable to develop a new model mouth system that can simulate the condition of buccal cavity as closely as possible, to determine the release of nicotine from moist snuff. The aims of the present study are to develop a model mouth system that can give a more realistic *in vitro* assessment for the bioavailability of nicotine to moist snuff users, and to validate the system by comparing with results of nicotine release in human volunteers.

Any experimental research reported in the manuscript has been performed with the approval of an appropriate ethics committee. Research carried out on humans must be in compliance with the Helsinki Declaration. In our manuscript, these STPs are commercial products. The study subjects filled out consent forms before the study started, which was stated in our manuscript.

### Experimental

#### Chemicals and materials

Nicotine was purchased from Acros Oganics (New Jersey, USA). HPLC grade methanol, triethylamine, phosphoric acid, and KH_2_PO_4_ were purchased from Merck (Darmstadt, Germany). Mucin from bovine submaxillary gland, α-amylase from human saliva, lysozyme from chicken egg white, and acid phosphatase from potato were obtained from Sigma (Missouri, USA). Analytical grade Urea and glucose were obtained from Kaitong Chemical Reagent Company (Tianjin, China).

Artifical saliva was prepared according to calculated amounts based on Chou and Hee [18]. It was composed of NaCl (1.4 mg/mL), KCl (0.5 mg/mL), CaCl_2_ (0.1 mg/mL), NaH_2_PO_4_ (0.15 mg/mL), MgCl_2_ (0.025 mg/mL), urea (0.09 mg/mL), glucose (0.2 mg/mL), mucin (2.7 mg/mL), α-amylase (2.5 units/mL), lysozyme (0.7 units/mL), acid phosphatase (0.004 units/mL). The solution pH was adjusted to 7.0 before addition of proteins.

Twenty-three brands of moist snuff (Table 1) manufactured by twelve different companies were selected for analysis. Eleven of them were purchased in 2010 from retailers in the United States and others were purchased from Internet retailers in 2010. Among these moist snuff products, the General Portion (GP) and Ettan Portion (EP) Snus were the two brands used for model mouth system development and validation. Moist snuffs were stored in a freezer (−18°C) until the analysis.

**Table 1:** Information of 23 selected moist snuff products for nicotine release analysis (mean ± SD, n = 5)

**Number**	**Brand variety**	**Manufacturer**	**Weight (g/pouch)**	**pH**	**Nicotine (mg/g,wet weight)**	**Free-based nicotine (mg/g,wet weight)**
1	Skruf Tranb	Skruf Snus	0.92 ± 0.05	7.36	9.60 ± 0.08	1.72
2	Skruf Stark	Skruf Snus	0.95 ± 0.03	8.01	11.09 ± 0.13	5.48
3	Skruf Xtra Strong	Skruf Snus	0.97 ± 0.04	8.10	14.21 ± 0.21	7.76
4	Gotland Fläder (green)	Swedish Match	0.94 ± 0.03	7.71	8.43 ± 0.11	2.77
5	Göteborgs Rapé White	Swedish Match	1.07 ± 0.03	7.84	5.78 ± 0.04	2.30
6	Ettan	Swedish Match	0.98 ± 0.03	8.32	5.95 ± 0.05	3.96
7	General	Swedish Match	0.98 ± 0.02	7.36	6.41 ± 0.07	1.15
8	General White	Swedish Match	0.96 ± 0.03	7.80	5.59 ± 0.07	2.10
9	Catch White Eucalyptus	Swedish Match	0.96 ± 0.02	7.81	5.71 ± 0.08	2.18
10	Jakobssons Wintergreen	Gotlands Snus AB	0.95 ± 0.02	8.98	5.41 ± 0.05	4.88
11	Thunder Xtra Strark	Gotlands Snus AB	0.90 ± 0.04	8.60	16.32 ± 0.15	12.92
12	Skoal Berry Blend	US Smokeless Tobacco Company	1.54 ± 0.02	7.52	9.98 ± 0.11	2.40
13	Copenhagen Original	US Smokeless Tobacco Company	1.40 ± 0.04	7.70	9.51 ± 0.10	3.08
14	Kodiak Premium wintergreen	American Snuff Company, LLC	1.43 ± 0.04	7.61	9.62 ± 0.09	2.69
15	Grizzly wintergreen	American Snuff Company, LLC	1.40 ± 0.05	7.96	9.67 ± 0.10	4.50
16	Klondike Peppermint blast	Nordic American Smokeless Inc.	0.52 ± 0.03	6.41	11.00 ± 0.08	0.26
17	Longhorn Straight	Pinkerton Tobacco Co.LP	0.94 ± 0.03	7.51	11.45 ± 0.23	2.70
18	Timber Wolf Packs Peacht	Pinkerton Tobacco Co.LP	1.54 ± 0.04	7.56	12.23 ± 0.24	3.15
19	Renegades Wintergreen	Pinkerton Tobacco Co.LP	1.02 ± 0.03	7.56	10.86 ± 0.33	2.80
20	Camel winterchill	R.J. Reynolfs Tobacco Company	1.03 ± 0.05	7.83	6.52 ± 0.09	2.56
21	Marlboro Peppermint	PHILIP MORRIS	0.51 ± 0.03	6.65	9.83 ± 0.15	0.40
22	Discreet Emerald ice	American Smokeless Tobacco Co. LLC	0.36 ± 0.01	5.80	15.37 ± 0.31	0.09
23	Kundli(Haryana)	Harsh International Khaini Pvt. Ltd.	0.36 ± 0.02	10.24	4.77 ± 0.09	4.74

### Design of a mode mouth system

**Figure 1:**
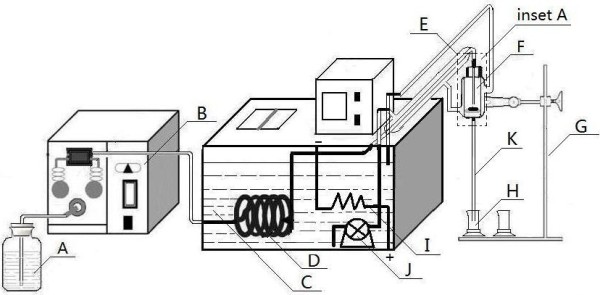
**Schematic diagram of the model mouth system.** Release medium reservoir **(A)**, constant flow pump **(B)**, water bath **(C)**, pre-warming coils **(D)**, water jacket tubing **(E)**, release cell **(F)**, release cell support bracket **(G)**, and fraction collector **(H)**, heating wire **(I)**, circulating pump **(J)**, collection tube **(K)**.

**Figure 2:**
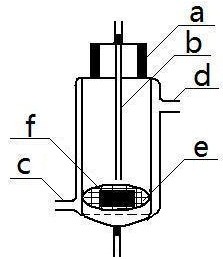
**Schematic diagram of the release cell (inset A).** Glass cap **(a)**, release medium supply tube **(b)**, water jacket inlet **(c)**, and water jacket outlet **(d)**, sample holding mesh **(e)**, moist snuff sample **(f)**.

A model mouth system (Figure 1) made in our laboratory was employed for *in vitro* analysis of oral STPs, which consists of a release medium reservoir (A), a constant flow pump (B), a water bath (C), pre-warming coils (D), water jacket tubing (E), the release cell (F), release cell support bracket (G) and a fraction collector (H). The release medium reservoir was a brown glass bottle. The constant flow pump was a HPLC dual piston pump with the flow accuracy ± 2% and the flow precision ± 1%, which provided constant flow rates from 0.1 to 10.0 mL min^-1^. The water bath temperature scale ranged from room temperature plus 5 to 95°C. The temperature fluctuation of the water bath was ± 0.1°C. The pre-warming coils, manufactured from silicon tube with an inner diameter of 1.0 mm and a length of 10 m, was placed in the water bath. The release medium tube between the water bath and the release cell was wrapped with glass water jacket tubing, with an outer diameter of 3 cm and an inner diameter of 4 mm. The interlayer of water jacket tube was connected with the water bath and the release cell (Figure 2). The release cell is a columniform glass cannula with 8 cm length, 4 cm inner diameter, and 6 cm outer diameter. The lower part of the release cell had a sample holding mesh (e). The glass cap plug had a glass catheter (b) with an inner diameter of 1.0 mm. The release cell was transparent which facilitated visual inspection during the operation. The fraction collector consisted of a tray and a number of flasks.

### Measurement of pH and total nicotine in STPs

Two grams of moist snuff were suspended in 20 mL deionized water and shaken for 15 min. The pH of the supernatant solution was then measured with a combination electrode as follows the reported method [19].

One gram of moist snuff was suspended in 50 mL mixed solution of ethanol and 1.25 M NaOH solution (9:1, v:v) and sonicated for 30 min in an ultrasonic bath(VWR Signature Model 75D, 90 W, USA). The extract was analyzed by liquid chromatography (LC) to obtain the total amount of nicotine in the moist snuff. The content of nicotine in the free-base form was calculated from the Henderson-Hasselbalch equation.

### *In Vivo* release of nicotine in STPs

*In vivo* release test of nicotine from two Sweden Snus (GP and EP) was carried out by 15 healthy volunteers, male, aged 27–48, according to study protocol. The study subjects filled out consent forms before the study started. All of them have a smoking experience but no STPs. No STPs and cigarette were used with one hour prior to study commencement. No food or drink during the trial other than option for water between Snus uses. All samples of moist snuff used for the study were selected at the same time from one container. Prior to the test, all volunteers were asked to rinse their mouth with distilled water. The sample was placed between the upper jaw and gum, and immediately a chronometer was started. Moist snuff was held in-mouth by subjects for 5 min, 10 min, 20 min, or 30 min, respectively, and the remaining amount of nicotine in each used sample was extracted with ethanol/NaOH solution and analyzed by LC. The amount of *In vivo* released nicotine was determined by the total nicotine amount in unused sample minus the remaining nicotine amount in used one. The release percentage was calculated by dividing the amount of *in vivo* released nicotine by the quantity in unused Snus multiplied by 100. The average of the triplicate measurements was performed for each Snus sample.

### *In Vitro* release of nicotine in STPs

*In vitro* release test was carried out by the home-built mode mouth system (Figure 1) using artificial saliva as a release medium. Firstly, artificial saliva was poured into the release medium reservoir (A). When the model mouth system was preheated to 37°C by the circulating water, a pouch of STPs was carefully placed on the sample holding mesh (e) on the lower part of the release cell (F), and then turn on the constant flow pump, artificial saliva flowed from the release medium supply tube (b) which was left about 2 mm above the pouch, and soaked the pouch. The exudate from the soaked pouch was collected in the fraction collector (H) and directly analyzed for nicotine by LC. Artificial saliva was heated while it flowed through the pre-warming coils and the warm water tube. The temperature of artificial saliva was equilibrated to 37°C before reaching the release cell.

The *in vitro* release test run lasted for 60 min, and the exudate from the soaked pouch was collected at 5-min intervals. After each run, the moist snuff sample was taken out from the release cell and placed into a conical flask. And then 50 mL of mixed solution of ethanol and 1.25 M NaOH solution was added to the flask and sonicated for 30 min in an ultrasonic bath. The extract was analyzed for nicotine by LC. The cumulative release percentage of nicotine at a time period was calculated by dividing the amount of the released nicotine by the total nicotine (released nicotine plus residual nicotine) and multiplied by 100. The release cell was cleaned with deionized water after each use.

### Liquid chromatography analysis

Chromatography analysis of nicotine in sample was performed on an Agilent 1200 series high performance liquid chromatography system equipped with a diode array detector (190–400 nm). The chromatographic data were processed with Agilent chromatographic Work Stations software.

The chromatographic separation was accomplished on a Waters XTerra RP C18 Column (250 mm × 4.6 mm i.d., 5 μm). The column temperature was maintained at 35°C. The mobile phase used for the separation was a 23:76.8:0.2 (v/v/v) mixture of MeOH, Potassium dihydrogen phosphate (20 mM, pH 6.0) and Triethylamine, delivered at a flow rate of 1.0 mL min^−1^ with an injection volume of 10 μl. The detection wave length was set at 260 nm. Before injection, all samples were filtered through a 0.45 μm membrane filter.

### Statistical analysis

Data were expressed as the mean ± standard deviation (SD) of replicate determinations (n ≥ 3). Statistical analyses were performed using SPSS v16.0 (SPSS Inc., Illinois, USA). Significance of differences in means of paired samples was assessed using paired-sample t-test. Correlations between the nicotine release rates and moist snuff unit dose weights, nicotine concentration in the pouch, and product pH values were tested with the Pearson correlation test. P values were based on two-tailed tests and P values of less than 0.05 were considered statistically significant.

## Results and discussion

### LC analysis of nicotine sample from STPs analysis of nicotine in sample was performed by LC

The LC assay method was validated for linearity, limits of detection (LOD) and limit of quantitation (LOQ), precision (inter-day and intra-day precision), accuracy, and stability of the nicotine extraction solution following the International Conference on Harmonization (ICH) guideline [20].

#### Linearity, LOD, LOQ, precision, and accuracy

Nicotine stock solution in methanol was prepared and diluted to appropriate concentrations for construction of the calibration curves. The standard solutions of nicotine over the range 5–600 μg mL^−1^ at 8 different concentrations were injected in triplicate. A linear regression equation (y = 10.6049 x - 8.4364) was constructed by plotting the peak areas versus the concentrations of nicotine with a regression coefficient of 0.9999. LOD and LOQ of the assay were determined based on the signal/noise (S/N) criteria and were found to be 0.20 μg mL^−1^ (S/N = 3) and 0.65 μg mL^−1^ (S/N = 10), respectively. In all cases, the LOD and LOQ were significantly below the levels of nicotine determined in the test samples.

Intra-day precision was determined from the analysis performed on the same day of eight independent replicates at low, medium and high levels of nicotine and inter-day precision was calculated on five independent replicates tested over five days. The intra- and inter-day precisions were expressed as the relative standard deviation (RSD). RSD ranged from 0.32% to 0.53% and 0.65% to 1.08% for inter-day and inter-day measurements, respectively, indicating excellent assay precision (Table 2).

**Table 2:** Intra-day and inter-day precision of nicotine analysis

**Concentration (μg mL**^**−1**^**)**	** Intra-day**^**a**^	** Inter-day**^**b**^
	**RSD (%,n = 8)**	**RSD (%,n = 5)**
28.33	0.32	0.65
118.67	0.47	0.76
458.26	0.53	1.08

^a^Intra-day precision in 1 day from eight analysis.

^b^Inter-day precision of 5 analysis on 5 different days.

Accuracy of the LC nicotine analysis method was assessed by the standard addition method. Nicotine standards were added to the known sample (GP, batch no.7) at three different levels and then extraction and analysis were performed as described in experimental section. The results of nicotine recovery rates are presented in Table 3. The percent of nicotine recovery ranged from 97.52% to 102.37% and the RSD values were less than 2.04%.

**Table 3:** Nicotine recovery tests to evaluate assay accuracy (n = 6)

**Original (mg g**^**−1**^**)**	**Spiked (mg g**^**−1**^**)**	**Measured (mg g**^**−1**^**)**	**Recovery (%)**	**RSD (%)**
6.41	2	8.38	97.52	2.04
	6	12.24	102.37	1.37
	15	20.96	98.58	1.13

Recovery (%) = [(measured amount - original amount)/spiked amount] × 100.

#### Stability of the nicotine extraction solutions

The stability of nicotine extraction solutions from STPs was evaluated at room temperature. The samples at three nicotine levels (28.33, 118.67 and 458.26 μg mL^−1^) were analyzed in triplicate every 8 hrs within 24 hrs. In all cases, peak shapes and retention times of nicotine were not affected, no additional peaks were detected, and no changes in the chromatographic pattern were observed. The RSDs at three levels of nicotine were 0.96%, 0.66%, 0.43%, respectively. These results indicate the solutions of nicotine extraction/release were stable within 24 hrs.

### The model mouth system

#### Theory

Oral environment was affected by many factors, of which saliva compositions, secretion rate of saliva, and buccal temperature are main factors to affect the release behavior of components in SPTs. In this paper, a model mouth system (Figure 1) was developed to simulate the conditions of oral cavity.

The temperature control module, which consists of a water bath, pre-warming coil and water jacket/tubing, was capable of adjusting the temperature of the model mouth system. The pre-heating coil, through which artificial saliva flowed, was immersed into a water bath. The interlayer of the water jacket/tubing, the interlayer release cell and the water bath were connected by pipe lines to maintain the release cell at a constant temperature. Driven by the constant flow pump, the artificial saliva flowed through the temperature control module and was pre-heated. When the artificial saliva arrived at the release cell, its temperature can reach the required temperature.

The release cell was used as a simulation oral cavity. The interlayer of release cell, which was filled with water of the presetting temperature, can ensure the release cell is conditioned at desired temperature.

#### Simulation for conditions of oral cavity

As is known to all, saliva is a mixture of secretions from the parotid, submaxillary and sublingual glands. It is impossible to duplicate human saliva due to the inconsistent and unstable nature, and individual differences in composition. So, artificial saliva was often used to replace natural saliva in study. In the work, the artificial saliva reported by Chou and Hee [18] was employed to carry out our experiments, which is composed of mineral components and organic components of nature saliva. Mucin is one of the organic components, which can increase viscosity and as such influences the diffusion rate of nicotine. The ingredient was not further studied in the work and directly employed to simulate natural saliva due to our methodological intention.

Under normal physiological conditions, secretion rate of human saliva is in the range of 0.06–1.8 mL/min. However, in a maximal simulation of the parasympathetic system, it can be increased to more than 7.0 mL/min [21,22]. Therefore, the model mouth system is designed to adjust the flow rate of artificial saliva between 0.06 mL/min and 7.0 mL/min in order to simulate secretion of human saliva. Secretion rate of saliva can be simulated by adjusting the flow rate of artificial saliva.

**Figure 3:**
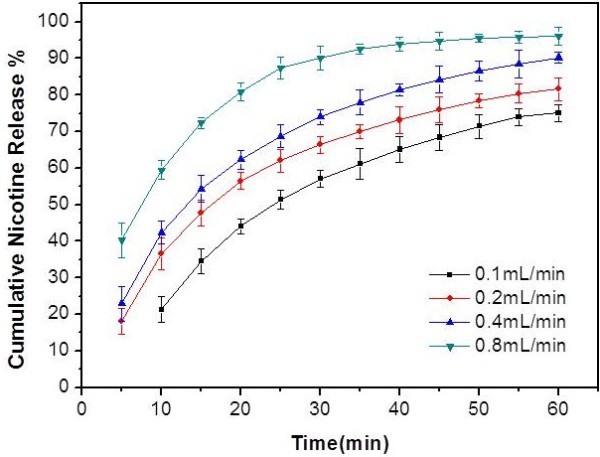
**Nicotine release profile of Sweden Snus GP at four different flow rates of artificial saliva.** The vertical bars represent the SD of five measurements.

The flow rate of artificial saliva was investigated at four different levels (0.1, 0.2, 0.4 and 0.8 mL/min). The test was performed at a constant temperature of 37°C using the sample GP. The release profiles of GP at the different flow rates were shown in Figure 3. It was notable that the flow rate of artificial saliva had a significant effect on nicotine release of GP (p < 0.05). These results indicated the stimulation intensity of moist snuff on oral cavity may have a significant effect on the release of nicotine.

**Figure 4:**
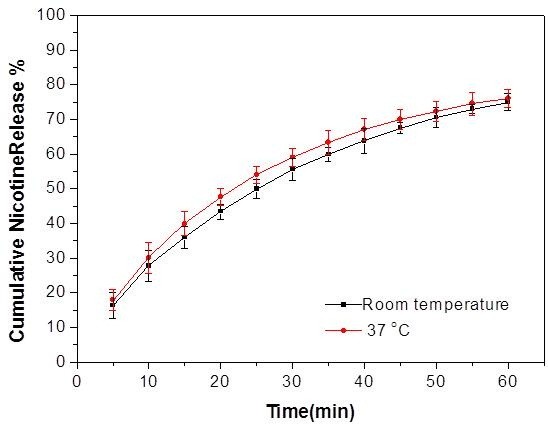
**Nicotine release profile of Sweden Snus GP at room temperature (25°C) and 37°C.** The vertical bars represent the SD of five measurements.

The temperature of the model mouth system can be maintained at a constant temperature through temperature control module. The influence of temperature on nicotine release from moist snuff was studied at room temperature (25°C) and 37°C. The test was performed at the flow rate of 0.2 mL min^-1^ in the first 5 min and 0.1 mL min^-1^ in the next 55 min using artificial saliva as the release medium. The release profiles of GP at each different temperature are shown in Figure 4. Results showed that nicotine release rate at 37°C were significantly faster than at room temperature (p < 0.05).

In this work, 37°C was selected as the temperature of the model mouth system because it is very close to the temperature of oral cavity in normal physiological conditions. The temperature was used to study *in vitro* release behavior of nicotine in STPs. Artificial saliva was heated while it flowed through the pre-warming coils and the warm water tube. The temperature of artificial saliva can be equilibrated to 37°C before reaching the release cell.

#### Validation of the model mouth system

To validate the model mouth system, nicotine release behavior of two Sweden STPs were obtained by in vivo test with healthy volunteers and *in vitro* test using the model mouth system, respectively.

#### In vivo nicotine release study

The cumulative nicotine release percentages from moist snuff GP and EP were assessed in 15 healthy volunteers. The cumulative nicotine release percentage of GP was 16.5 ± 4.1% (mean ± SD, ranged from 11.1% to 23.4%) at 5 min, 24.4 ± 4.5% (ranged from 18.4% to 31.5%) at 10 min, 34.2 ± 4.8% (ranged from 26.6% to 43.2%) at 20 min, and 44.2 ± 9.1% (ranged from 34.6% to 57.2%) at 30 min. For EP, The cumulative nicotine release percentage was 18.8 ± 5.9% (ranged from 10.4% to 29.1%) at 5 min, 29.0 ± 7.2% (ranged from 19.0% to 39.6%) at 10 min, 39.0 ± 8.4% (ranged from 24.7% to 49.8%) at 20 min, and 48.4 ± 6.1% (ranged from 38.5% to 59.4%) at 30 min.

**Figure 5:**
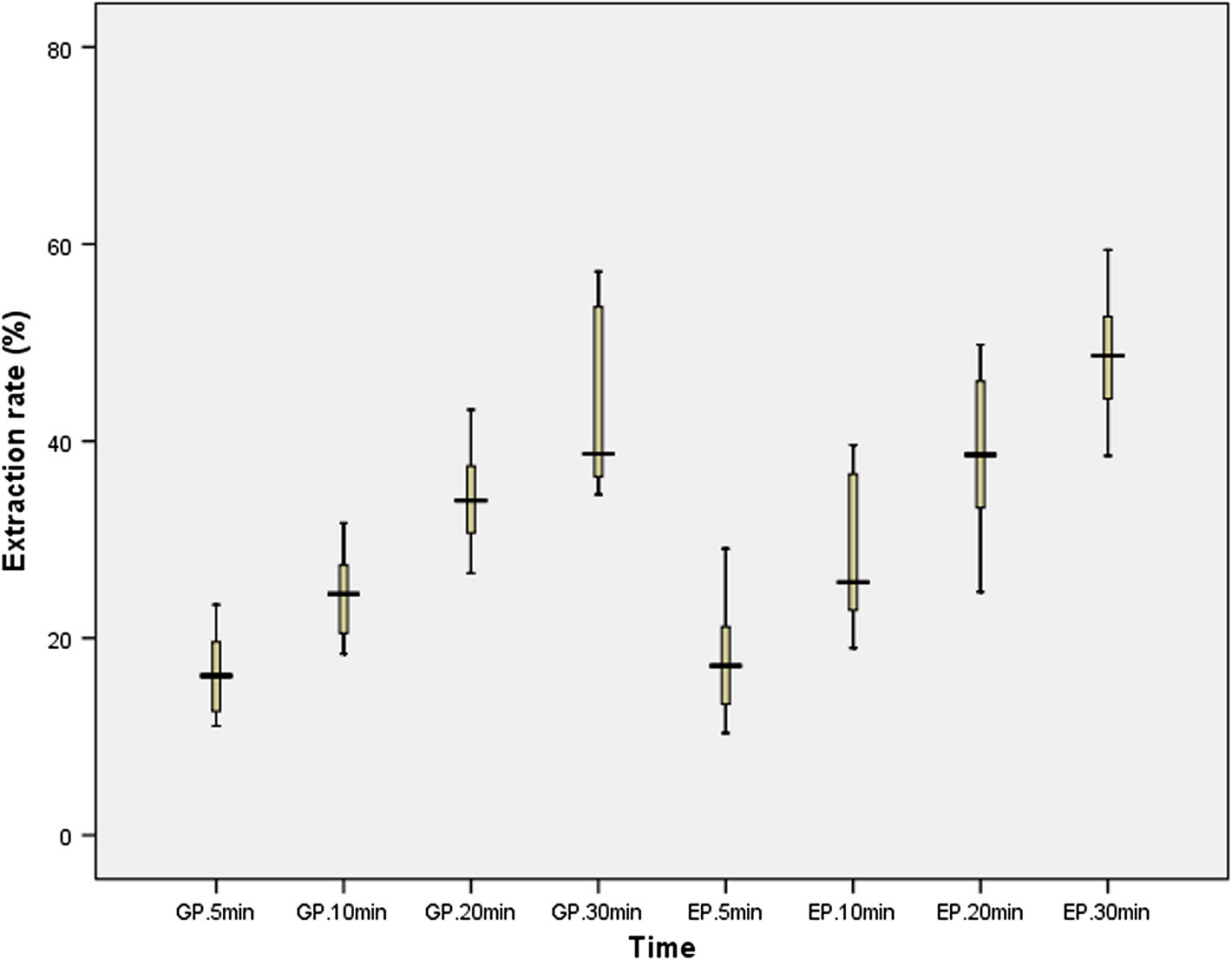
Boxplot of the results for the extraction rates of nicotine from two Snus by volunteers.

The cumulative nicotine release percentages of two STPs with 15 subjects are illustrated as boxplot in Figure 5. The values are bigger relative to the data of references [23-27]. The reason may be that the subjects in our study have no an experience used STPs. When they use the STPs, they maybe secrete larger amount of saliva than the experienced users of STPs, which causes more nicotine extracted from the STPs. There were significant inter-individual variations in the cumulative nicotine release percentages under the same experimental conditions, which indicated that it may be not an ideal approach to use human subjects as the first line assay for the evaluation and development of moist snuff products, not even mention the ethics and safety concerns of using human subject for STPs testing.

#### In vitro nicotine release studies

**Figure 6:**
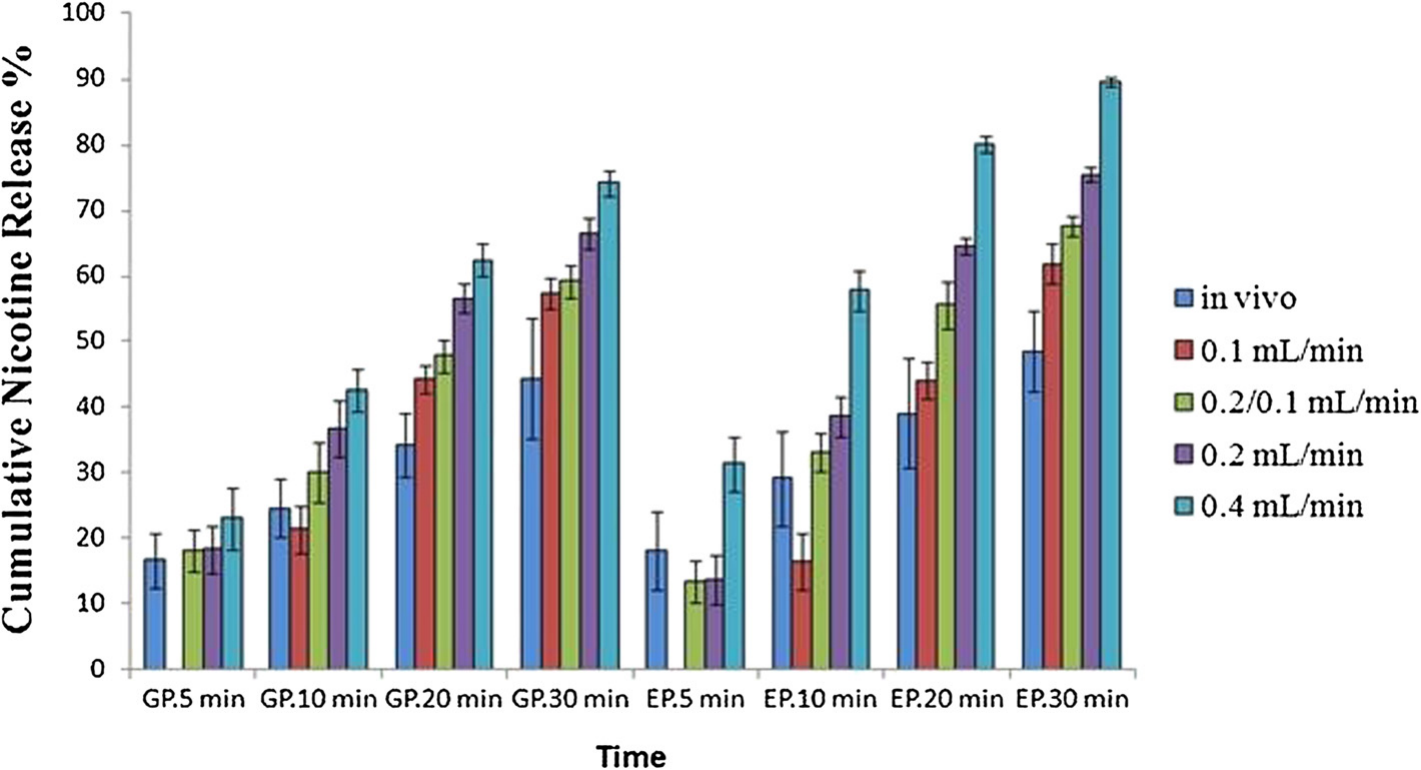
**Comparison of *in vivo *and *in vitro *nicotine release.***In vivo* nicotine release rates are mean ± SD of fifteen determinations. *In vitro* nicotine release values are mean ± SD of five determinations.

**Figure 7:**
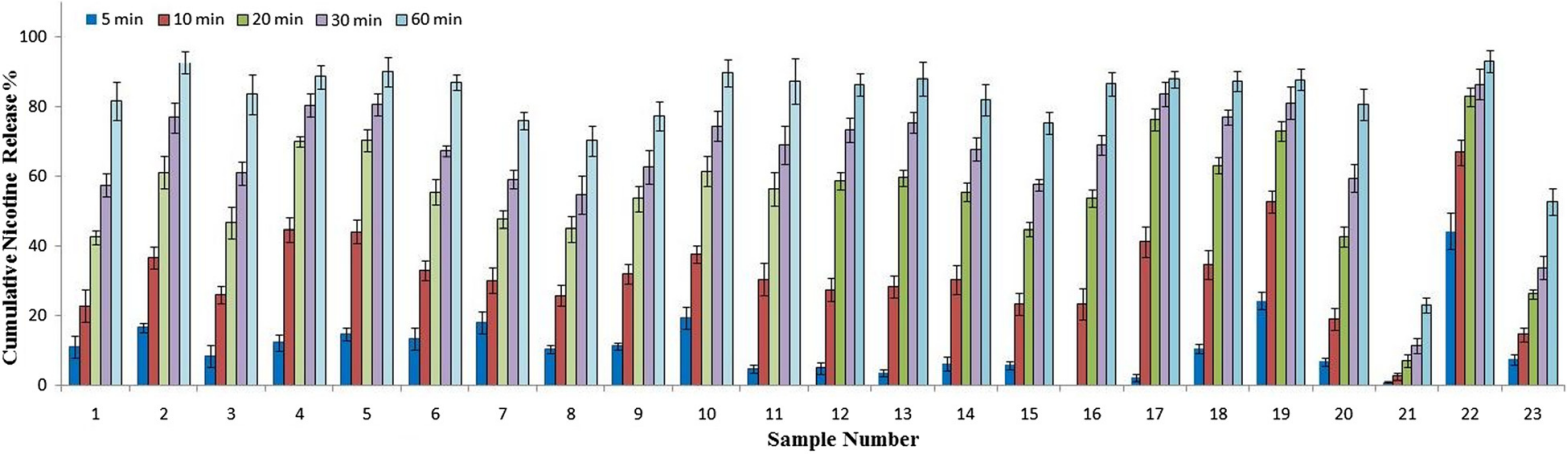
The cumulative nicotine release percentages from 23 brands of moist snuff.

*In vitro* release behavior of nicotine in STPs was simulated by the model mouth system according to the data of *in vivo* test. The flexibility of adjusting parameter settings on the model mouth system makes it possible to achieve good *in vitro* and *in vivo* correlations. Figure 6 shows a comparison of the cumulative nicotine release percentages of *in vivo* and *in vitro* test. The cumulative nicotine release percentage at lower flow rate (0.1 mL min^-1^) was a better match to human data; however, in both GP and EP, there was a seven to eight minutes moisture pickup process before nicotine release begins when the flow rate was at 0.1 mL min^-1^ (as shown in Figure 6, there was no nicotine release at 5 min with a flow rate of 0.1 mL min^-1^). In human subjects, the average cumulative nicotine release percentages of GP and EP after five minutes were 16.5% and 18.0%, respectively. Therefore, the flow rate was set to 0.2 mL min^-1^ in the first 5 min, and then it was changed to 0.1 mL min^-1^ after 5 min to better match *in vivo* nicotine release process. With the flow rate program of artificial saliva, we obtained the cumulative nicotine release percentages of GP and EP. The results (Figure 7) showed that the cumulative nicotine release percentages obtained by the model mouth system were similar to those by *in vivo* test for each a time terminal. This indicated that the developed method provides an alternative way to study release behavior of nicotine in STPs.

### Application of the model mouth system to evaluate nicotine release in 23 moist snuff products

Twenty three brands of moist snuff were analyzed in artificial saliva by the model mouth system. The weights, nicotine contents, pH, and free-based nicotine content of the selected samples for nicotine release analysis were determined (n = 5) and results were summarized in Table 1. The flow rate was 0.2 mL min^-1^ in the first 5 min, and then was changed to 0.1 mL min^-1^. The cumulative release rates of nicotine were shown in Figure 7. Nicotine release rates increased with extraction time and approximately 60-90% of nicotine was released after 30 min of extraction in most of the samples, which was consistent with the general suggested time of moist snuff use.

There were differences in nicotine release rates among the tested brands of moist snuffs, and the differences were significant for a couple of brands (Marlboro peppermint pack Snus and Kundli). We evaluated the impact of pouch weight, nicotine content, and the pH of each moist snuff product by correlating these parameters with nicotine release rates at 30 min of extraction using the Pearson correlation test. Nicotine release rates were positively correlated with pouch weight and pouch nicotine concentration, and negatively correlated with pH values; however, the overall correlations were weak, with correlation efficiencies (r) of < 0.36. Nicotine release rates in sample No. 21 (Marlboro peppermint pack Snus) were significantly slower than in other samples (Figure 7). When sample 21 pouches were cut open, it was noticed that there was a thin film in each pouch, which might have caused the slower release of nicotine from the sample. The slower nicotine release in sample No. 23 may be partially related to its high pH value (pH = 10.24).

## Conclusions

A novel model mouth system was developed and applied to evaluating the *in vitro* release behavior of oral use STPs. The operational parameters of the system, such as flow rate, extraction cell temperature, extraction solution chemistry composition, extraction time, etc., can be easily adjusted to fit different purposes. The system was validated to mimic conditions of human moist snuff consumption and applied to investigate the nicotine release profile in 23 brands of moist snuff products. Results indicated that the system performance is reliable, replicable, and consistent with human data under appropriate parameter settings. The model mouth system can be used to evaluate the release behavior of chemical constituents in moist snuff, especially those directly related to human health such as nicotine and tobacco specific nitrosamine, etc. With testing and validation, the model mouth system could be used as an alternative approach in risk evaluation of smokeless tobacco products.

## Competing interests

The authors declare that they have no competing interests.

## Authors’ contributions

PL and JZ co-participated in the experimental design of the study and performed chemical analysis. SHS coordinated the study, performed data analysis and drafted the manuscript. JPX and YLZ provided expert scientific advice and revised the manuscript. All authors read and approved the final manuscript.
